# Commentary to "Review of Internet Health Information Quality Initiatives"

**DOI:** 10.2196/jmir.3.4.e29

**Published:** 2001-12-26

**Authors:** Cynthia Baur, Mary Jo Deering

**Affiliations:** ^1^Office of Disease Prevention and Health PromotionU.S. Department of Health and Human ServicesUSA

The report of Ahmad Risk and Joan Dzenowagis for the World Health Organization [1] provides a useful compendium and commentary for those who are trying to get a handle on the proliferation of quality improvement mechanisms for the health Internet. The authors identify the prevention of harm and citizen protection as the main motivations for quality improvement efforts. We suggest that realizing the potential of the Internet for health improvements should be an equal if not more important reason to undertake quality improvement activities. This affirmative purpose is important because the current evidence suggests that actual harm has been negligible to date, and over time, benefits on a population basis could be substantial.

The report summarizes and comments on thirteen quality improvement initiatives that have been developed since the mid-1990s. Some are included mainly for historical purposes. Although the authors include a section on "implementation mechanisms," there is no clear means for someone who has not been following the progress of the initiatives to distinguish which initiatives are most substantial, furthest along and likely to be supported by health Internet Web sponsors. Readers would benefit from further categorization of initiatives in terms of potential for realization and likely support in the health Internet space.

The paper provides persuasive evidence that the commonalities of substance among the various approaches outweigh the differences of their specific implementation approaches. The authors observe that all the initiatives begin with quality criteria that derive from similar roots and have involved consensus-building, the scope of which depended on how the initiative's participants defined their interests and constituents. It is hard to avoid the conclusion that the time is ripe to bring the fundamental language of the different initiatives into harmony, and consolidate the agreement that has emerged to date. The author's equally persuasive discussion of the many remaining pitfalls in implementation and sustainability suggests that the individual initiatives might be interested in common solutions to share the burdens of operating and financing quality improvement activities on an on-going basis.

More consolidated implementation would also make it easier to think about how to deal with an issue that has been sidelined in much of the debate about quality standards: communication with end users about quality. Communication with users or the public broadly about the importance and nature of quality and quality standards has continuously been a missing but essential piece of the quality activities in the health Internet space. As the report acknowledges, the current crop of initiatives places a heavy burden on the end user to sort out what the criteria, seals, and tools mean and when to apply them. Consumer education about the importance of quality, the meaning of quality criteria and the different approaches merits a brief mention in the Recommendations, but it is clearly subordinated to the other activities listed in the section, despite the author's conclusion (#2) that an educated, interested and active citizenry is essential for the success of any quality program. Communicating with the public generally and end users of specific health Internet resources about quality must be a top priority for the field, no matter which initiatives survive and in what form.

The paper proposes that we need global leadership to move to the next generation of quality standards. Depending on the extent of Web site sponsors' participation, a global approach potentially decreases the proliferation of initiatives and consumer/citizen confusion about their different meanings and value. Global leadership could motivate a large number of Web sites to get behind the same set of criteria, or at least some core set. There is no question that being able to clearly communicate to users about a widely endorsed and recognizable set of quality standards, issues, and implementation would be a great public good. Although it would not be necessary that all public education about quality be coordinated by a single entity, global leadership in promoting culturally appropriate communications about core criteria and approaches would be beneficial. WHO is experienced in the varieties of health-related attitudes and practices and could extend this experience to the field of health information. But, global leadership and global governance are not the same. Global governance requires at least a minimum set of shared values and understanding of the problem and appropriate remedies, including the appropriate actors to undertake such actions. It is not clear yet that we have this in the health Internet space on a national, let alone international, scale.

Two problems clearly challenge any quality initiative: how to sustain it, and how to influence those health Internet actors who will most likely remain outside the boundaries of quality initiatives, especially those the author calls "pseudo health" and quackery. The authors carefully avoid recommending that WHO become the single guarantor of global standards, although they come close. Further clarification would be needed about the exact "what" and "how," before WHO could propose such a role to its member states. WHO would most likely have to make a long-term commitment and undertake activities outside its traditional mandate. Even more troubling, though, is the nagging certainty that even if the best system in the world were developed and implemented, it would most likely have its strongest influence on the better, more responsible, and more easily traceable Internet health activities. Those activities that are most spurious and likely to cause actual harm are also most likely to ignore quality standards, although clearly fraudulent activities may be covered by the laws of individual countries. Enforcement efforts in a few countries to date-and efforts from other sectors-would be instructive. In the U.S., a governmental apparatus exists to deal with fraud and quackery, although there is nothing comparable to address activities that do not rise to the level of law-breaking.

The time has come for a global dialog on Internet health quality and concrete steps toward harmonization, coordination, and-most important-effective communication to our many publics. We may not reach a fixed "solution" to the dual challenges of risks and benefits, but we could at least consolidate the path taken to date into a firm foundation for next steps.
